# Infrared Photoactivation Enables nano-DESI MS of Protein Complexes in Tissue on a Linear Ion Trap Mass Spectrometer

**DOI:** 10.1021/jasms.4c00377

**Published:** 2024-11-28

**Authors:** Oliver J. Hale, Todd H. Mize, Helen J. Cooper

**Affiliations:** School of Biosciences, University of Birmingham, Edgbaston, Birmingham B15 2TT, U.K.

## Abstract

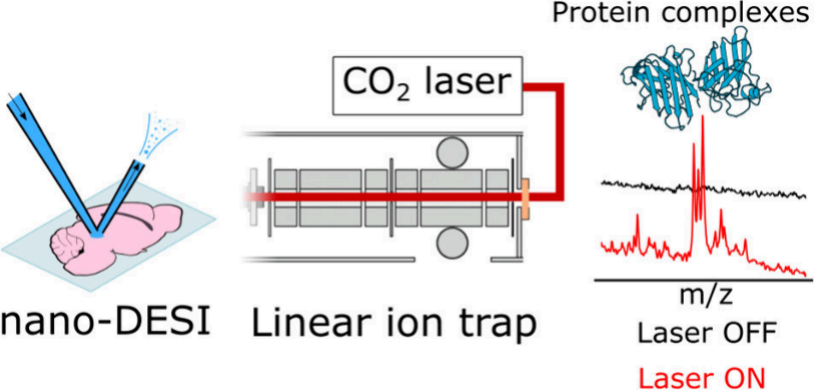

Native mass spectrometry analysis of proteins directly from tissues can be performed by using nanospray-desorption electrospray ionization (nano-DESI). Typically, supplementary collisional activation is essential to decluster protein complex ions from solvent, salt, detergent, and lipid clusters that comprise the ion beam. As an alternative, we have implemented declustering by infrared (IR) photoactivation on a linear ion trap mass spectrometer equipped with a CO_2_ laser (λ = 10.6 μm). The prototype system demonstrates declustering of intact protein complex ions up to approximately 50 kDa in molecular weight that were sampled directly from brain and eye lens tissues by nano-DESI. For example, signals for different metal binding states of hSOD1^G93A^ homodimers (approximately 32 kDa) separated by only approximately 6 Th (10+ ions) were resolved with IR declustering, but not with collisional activation. We found IR declustering to outperform collisional activation in its ability to reduce chemical background attributable to nonspecific clusters in the nano-DESI ion beam. The prototype system also demonstrates *in situ* native MS on a low-cost mass spectrometer and the potential of linear ion trap mass spectrometers for this type of analysis.

## Introduction

Native protein analysis directly from tissue by nanospray desorption electrospray ionization (nano-DESI) enables spatially resolved native top-down mass spectrometry (nTDMS), and mass spectrometry imaging (MSI) of protein complexes exceeding 100 kDa.^1,2^ So far, this methodology has been limited to high-performance mass spectrometers. The cost of high-performance mass spectrometers has the potential to be prohibitive to wider adoption of *in situ* native mass spectrometry in structural biology applications and more affordable instrumentation is a request of the broader native MS community.^3^ One factor that limits native MS performance is the need for declustering, i.e., removal of solvent, salts, and detergent from protein ions without disrupting noncovalent interactions in order to obtain accurate mass measurements.^4,5^ The ubiquity of collisional activation means it is often used for declustering in native MS experiments.^6−9^ The associated elevation of ion kinetic energy complicates transmission of the ions in the mass spectrometer and can be detrimental to sensitivity without further tuning, e.g., increasing trapping gas pressure^8,10,11^ or ion optics voltages.^7,12,13^ Each of these adds an additional level of complexity, requiring extra pumping capacity or more sophisticated ion optic elements.

Infrared multiphoton dissociation (IRMPD) is a charge-independent alternative to collision-induced dissociation (CID), that has shown promise for declustering ions from the chemical background and adducts.^14^ Brodbelt and co-workers demonstrated increased peptide and protein ion signal intensity after IRMPD of nonspecific chemical signal on a custom linear ion trap (LIT) mass spectrometer.^15^ Their study indicated that proteins incorporated in salt/solvent clusters could be recovered as intact protonated molecular ions. Separately, several studies have combined native MS and IRMPD-equipped ion trap mass spectrometers to fragment protein complexes^16−19^ and release membrane proteins from detergent micelles (with subsequent *m*/*z* analysis by TOF or orbitrap mass analyzers).^17−19^

Our recent work has focused on the use of nano-DESI under native-like conditions for the analysis of intact protein complexes directly from tissue sections. Native nano-DESI produces extremely heterogeneous ion beams from an electrospray containing intact proteins and protein complexes (i.e., the analytes of interest) mixed with nonspecific clusters of solvent, salts, detergent micelles, and abundant endogenous biomolecules (e.g., lipids) leading to substantial nonspecific chemical signal across a broad *m*/*z* range. Currently, collisional activation in the ion source and/or dedicated collision cell is used to release protonated protein complexes from the clusters. Although beneficial, native nano-DESI spectra still suffer from chemical background and low signal abundance with this approach, and to date, it has not been possible to transfer native nano-DESI to simpler, lower cost mass spectrometers that feature less sophisticated ion optics.

Here, we demonstrate that the nano-DESI ion beam can be declustered by IRMPD, allowing the release of intact protein ions and enabling native protein analysis from tissue on a LIT mass spectrometer. We attached our home-built nano-DESI ion source^20^ to a LIT mass spectrometer modified with a continuous wave infrared laser (CO_2_, λ = 10.6 μm). We observed a dramatic reduction in chemical background in nano-DESI spectra with IR activation of the ion beam compared with inactivated and collisionally activated ion beams. Our results suggest that IR declustering is a promising alternative to collision-based declustering for native protein analysis by nano-DESI from complex biological environments.

## Materials and Methods

### Materials

MS-grade water was purchased from Fisher Scientific. HPLC-grade ammonium acetate was bought from J.T. Baker (Deventer, The Netherlands). C_8_E_4_ detergent, polypropylene glycol, ubiquitin, and carbonic anhydrase 2 were obtained from Merck (Gillingham, UK). Helium gas (99.996% purity) was obtained from BOC (Guildford, UK). Calmix calibration solution was purchased from Thermo Fisher Scientific (Waltham, MA).

### Animal Tissues

Fresh frozen brains from SOD1G93A C57BL/6 transgenic mice were the gift of Dr. Richard Mead (University of Sheffield, UK).^21^ Each brain was bisected down the midline, and the left hemisphere was mounted to a chuck with ice. Sagittal cryosections were prepared by cutting from the midline with a CM1810 Cryostat (Leica Microsystems, Wetzlar, Germany) and thaw-mounted to glass microscope slides before storage at −80 °C until analysis.

Whole, fresh sheep eyes were bought from DissectUK (Birmingham, UK). Eyes were harvested and transported in cold packs for dissection. Lenses were extracted from each eye, placed on aluminum foil, and snap frozen in liquid nitrogen. All tissue was stored at −80 °C, sectioned at −22 to −24 °C to a thickness of 20 μm with a CM1810 Cryostat, thaw mounted to glass microscope slides, and stored at −80 °C until analysis.

### Linear Ion Trap Modification for IRMPD

An LTQ Velos Pro linear ion trap (LIT) mass spectrometer with a scan range up to *m*/*z* 4000 (Thermo Fisher Scientific, San Jose, CA) was recovered from a decommissioned Orbitrap Elite mass spectrometer (Thermo Fisher Scientific). A CF flange containing an IR-transparent ZnSe window (Thorlabs, Newton, NJ) was attached to the rear of the LIT. A continuous wave CO_2_ IR laser (λ = 10.6 μm; max power; 20 W, model; FireStar V20, Synrad Inc., Mukilteo, WA), an inline red diode pilot laser, and beam optics were recovered from a decommissioned LTQ-FT mass spectrometer (Thermo Fisher Scientific) and positioned at the rear of the LIT (see Figure S1, Supporting Information). With the LIT chamber open to the atmosphere, the pilot laser beam was transmitted through the LIT low-pressure (LP) cell and high pressure (HP) cell for initial beam alignment. The raw 10.6 μm output (2.0 mm × 2.4 mm elliptical cross section, 7 mrad divergence) was guided approximately 300 mm to the 2 mm diameter end orifice of the LIT using nonfocusing mirrors and ZnSe window, and alignment with the ion beam axis was ensured using 3 more LIT orifices as apertures and optimizing the beam transmission through all of these; transmission of the beam after the apertures was ^∼^20%. Transmission of the 10.6 μm beam was confirmed by heat-sensitive cards (Thorlabs) placed in the beam path at atmospheric pressure and by the observation of fragmentation of caffeine/MRFA/ubiquitin ions with the system under vacuum. IRMPD fragmentation of a protein complex was tested by infusing carbonic anhydrase 2 (10 μM in 200 mM aqueous ammonium acetate) in complex with zinc at a rate of 3 μL/min by electrospray ionization. The CO_2_ laser was triggered by an RSDG 805 arbitrary waveform generator (RS Group, London, UK) enabling laser output power control between 20% and 80% of maximum output power (4–16 W). The laser was operated continuously during ion accumulation (see Results and Discussion for accumulation times).

### Nanospray-Desorption Electrospray Ionization (nano-DESI) MS

A home-built nano-DESI ion source^20^ was attached to the atmospheric pressure interface of the LTQ Velos Pro. The solvent system was aqueous ammonium acetate (200 mM) + 0.125% (by volume) of the detergent C_8_E_4_ and set to a flow rate of 1.7 μL/min by an external syringe pump. Electrospray voltage was set to 1.15 kV. The ion inlet temperature was 275 °C, and the S-lens was set to 70%. Source pressure was approximately 1.5 Torr, and LIT pressure was approximately 1.7e-5 Torr (separate pressure readbacks for each cell were not available). Helium was provided to the HP cell as the damping gas. Tissue sections were continuously sampled by scanning under the nano-DESI probe at a rate of 5 μm/s. Ions were accumulated and continuously irradiated by 10.6 μm photons along the beam path through the high-pressure cell and transfer optics (see Figure S1, Supporting Information). The automatic gain control (AGC) target was set to 5e4 charges with a maximum ion accumulation (“injection”) time of 750 ms. Long injection times were necessary because of the low protein ion flux from direct tissue sampling, and typically the AGC target was not reached before injection. This requirement is also typical of these experiments on high-end Orbitrap systems. Ions were injected into the low-pressure cell for *m*/*z* analysis. Unless otherwise noted, the ion trap mode was set to “High Mass” and scan rate was set to “Turbo” (125000 (*m*/*z*)/s, peak fwhm; 3) with two microscans averaged per scan. Mass calibration was performed with CalMix and PPG 2700. Mass spectra were externally recalibrated to nano-DESI spectra recorded using an Orbitrap mass spectrometer (Orbitrap Eclipse, see below).

The same nano-DESI source was attached to an Orbitrap Eclipse (Thermo Scientific) equipped with the HMR^n^ option, as previously described,^1^ and setup as for the LTQ unless otherwise noted here. The electrospray voltage was set between 0.9 and 1.4 kV, and the ion inlet temperature was 275 °C. The S-lens was set to 120%, and in-source CID potential was 80 V with a scaling factor of 3%. Acquisition mode was set to “High Mass”, “Intact Protein”, “High Pressure” (ion routing multipole pressure = 20 mTorr, ion trap high pressure cell = 3.5e-5 Torr) and selected ion monitoring (SIM, ion trap isolation *m*/*z* 3200 ± 800). The AGC target was set to 10,000% (5e6 charges) with a maximum injection time of 750 ms, and the resolution setting was 7500 fwhm at *m*/*z* 200. The system was calibrated with a FlexMix (Thermo Fisher Scientific). For nano-DESI top-down MS^n^ experiments, the orbitrap resolution was set up to 500000 fwhm at *m*/*z* 200. Proteins were identified through a combination of MS/MS, deconvolution, and high-resolution MS.

## Results and Discussion

### Nano-DESI-IRMPD MS of Protein Complexes in Brain Tissue

Our previous work using nano-DESI for native MS of proteins from tissue has required high-end mass spectrometers for reasons including their high mass resolving power, ion/ion reactions, and ion mobility separation.^1,22^ An instrument suitable for native MS does not necessarily require a diverse set of functionality if its primary role is intact mass analysis, for example, which piqued our interest in using the LTQ Velos Pro. We have found it necessary to use collisional activation on high-performance instruments to generate declustered protein ions with usable signal quality. As a testbed for IR declustering on a lower cost instrument, we modified the LIT mass spectrometer to enable continuous irradiation of the ion beam during ion accumulation in the high-pressure cell, thereby declustering protein complexes from the solvent, detergent, and salts before *m*/*z* analysis. Overall, there was a stark improvement in mass spectra obtained with IR activation, which promises to enable native MS from challenging sample environments on lower cost systems.

**Figure 1:**
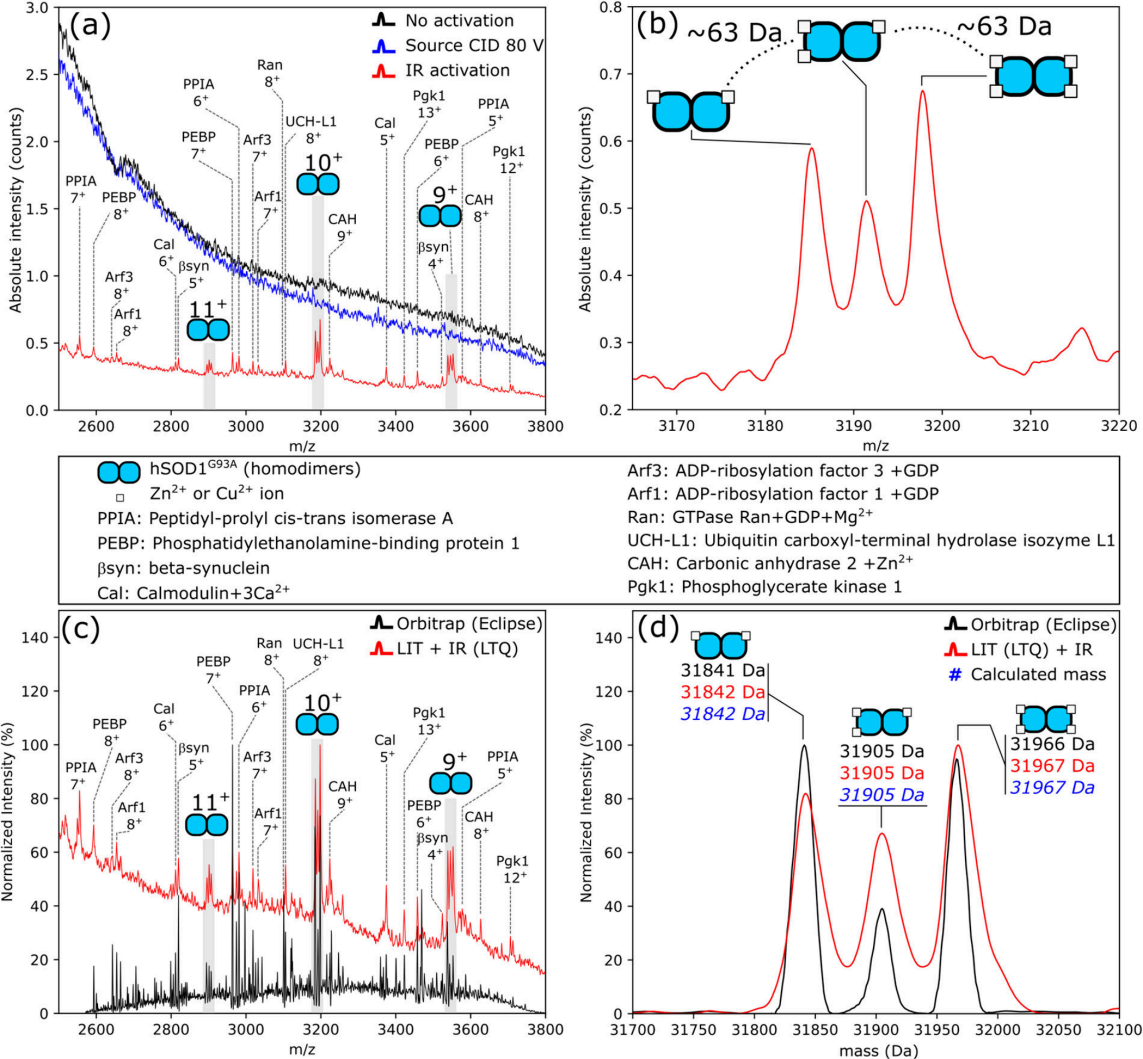
Nano-DESI mass spectra of transgenic mouse brainstem tissue. (a) Analysis using the prototype IR-LIT platform: laser off (black trace), with source CID (80 V, blue trace), and with laser output power approximately 5.6 W (red trace). (b) A distinctive triplet of signals, corresponding to endogenously occurring hSOD1^G93A^ homodimers in metal-deficient (two and three metal ions) and holo (four metal ions) forms (charge states 11+, 10+, and 9+). (c) Intensity-normalized nano-DESI mass spectra acquired on the Orbitrap Eclipse platform; orbitrap analyzer (black trace, resolution setting = 7500 fwhm at *m*/*z* 200, maximum intensity 1.25e4), and on the IR-LIT platform; linear ion trap (red trace, maximum intensity 6.78e-1). (d) Deconvoluted mass spectra of hSOD1^G93A^ complexes using mass spectra from orbitrap (black) and LIT (IR-LIT, red). The calculated mass for each complex is shown in blue (additional information is given in Table S3, Supporting Information).

The system was tested by spatially resolved nano-DESI analysis of protein–ligand and protein–metal complexes from the mouse brain. We sampled directly from the brainstem of a mouse brain tissue section from the G93A disease model of amyotrophic lateral sclerosis (ALS), in which a mutant form of the human protein SOD1 (hSOD1^G93A^) is expressed. Without ion activation and with in-source collisional activation, protein peaks were not detected (Figure 1a). With IR activation (5.6 W laser output power), the background chemical signal was reduced considerably, and protein signals could be detected (Figure 1a).

hSOD1^G93A^ exhibits irregular metal ion binding (i.e., dimers binding two and three metal ions) in addition to formation of the holo-form (binding four metal ions).^23^ The characteristic pattern of three peaks separated by approximately 63 Da in mass is observable in the nano-DESI-IR-LIT mass spectrum (Figure 1a,b, dimers in charge states 11+, 10+, and 9+). Other protein complexes in this tissue were also detected, including Arf3 and Arf1 (both molecular weights correspond to the protein in complex with their endogenous ligand guanosine disphosphate, GDP) and carbonic anhydrase 2 bound to its endogenous Zn^2+^ cofactor (molecular weights for detected brain proteins are given in Table S1, Supporting Information). Mass spectra from the IR-LIT correlate with nano-DESI spectra obtained from the same mouse model on an Orbitrap Eclipse using in-source CID for declustering and detection in the orbitrap mass analyzer (Figure 1c, Figure S2, Supporting Information). The Orbitrap Eclipse was developed with consideration for analysis of native MS of protein complexes.^12^ The orbitrap analyzer demonstrated higher signal intensity and low noise compared to the IR-LIT, in part owing to processing applied to the raw time-domain transient. The short time domain transient (here, transient duration = 16 ms) is beneficial for high signal-to-noise analysis of intact proteins at the expense of isotopic resolution and is typical for native MS analysis performed on these instruments.^24^ The IR-LIT also does not isotopically resolve the protein signals. Spectrum deconvolution of the hSOD1^G93A^ charge state envelope in Orbitrap and IR-LIT spectra was used to determine the intact mass of the complexes. Deconvolution of Orbitrap and LIT mass spectra was performed with UniDec^25^ (settings in Table S2, Supporting Information). The deconvoluted IR-LIT spectrum is comparable to the Orbitrap spectrum, with both having sufficient resolution to resolve the three metal-bound hSOD1^G93A^ complexes (Figure 1d, Table S3, Supporting Information).

### Native nano-DESI-IR-LIT MS of Proteins in Eye Lens Tissue

The eye lens features abundant soluble proteins, which form a variety of oligomeric complexes. The abundance of these complexes made them suitable to evaluate the effect of varying laser power. As with the brain tissue, protein ions were more effectively declustered by IR activation than with collisional activation and retain noncovalent interactions, and the chemical background was reduced.

**Figure 2:**
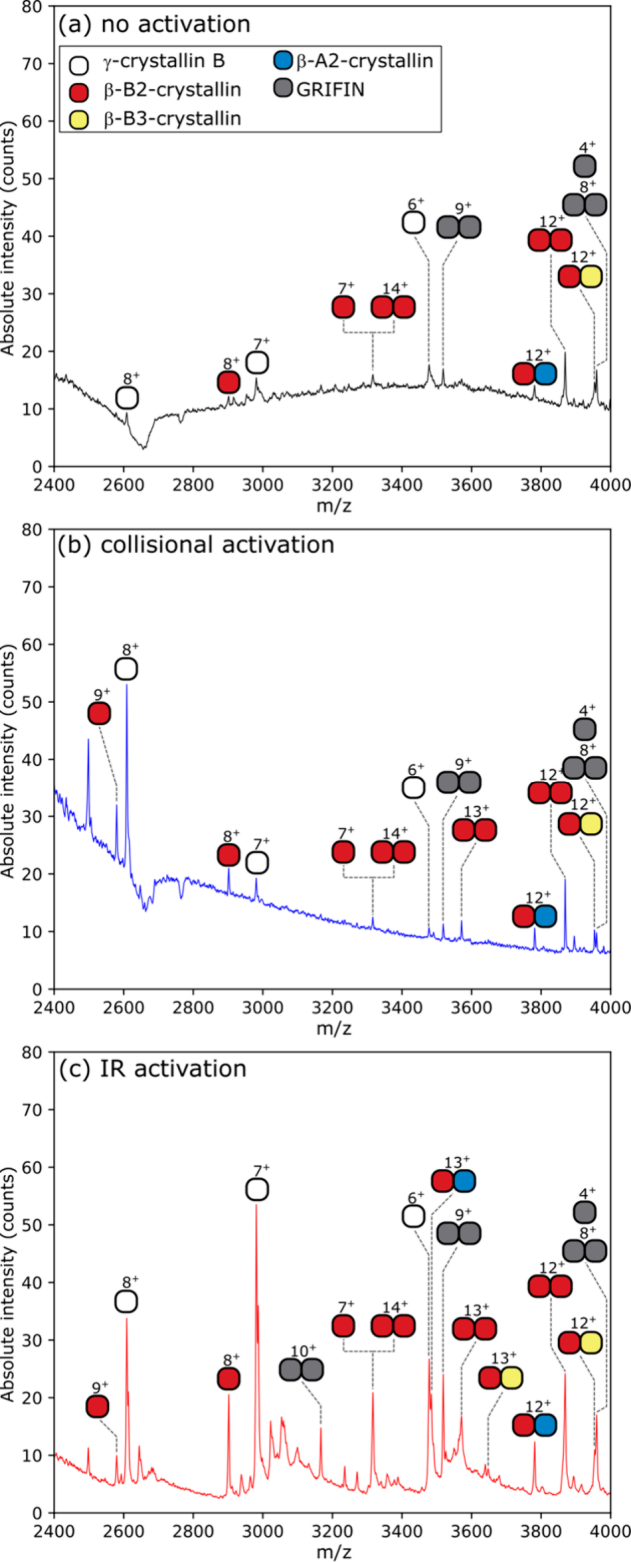
IR declustering of proteins sampled from sheep eye lenses by nano-DESI: (a) laser off, (b) with source CID = 100 V, and (c) with IR laser output power ∼6 W for up to 750 ms during ion accumulation.

**Figure 3:**
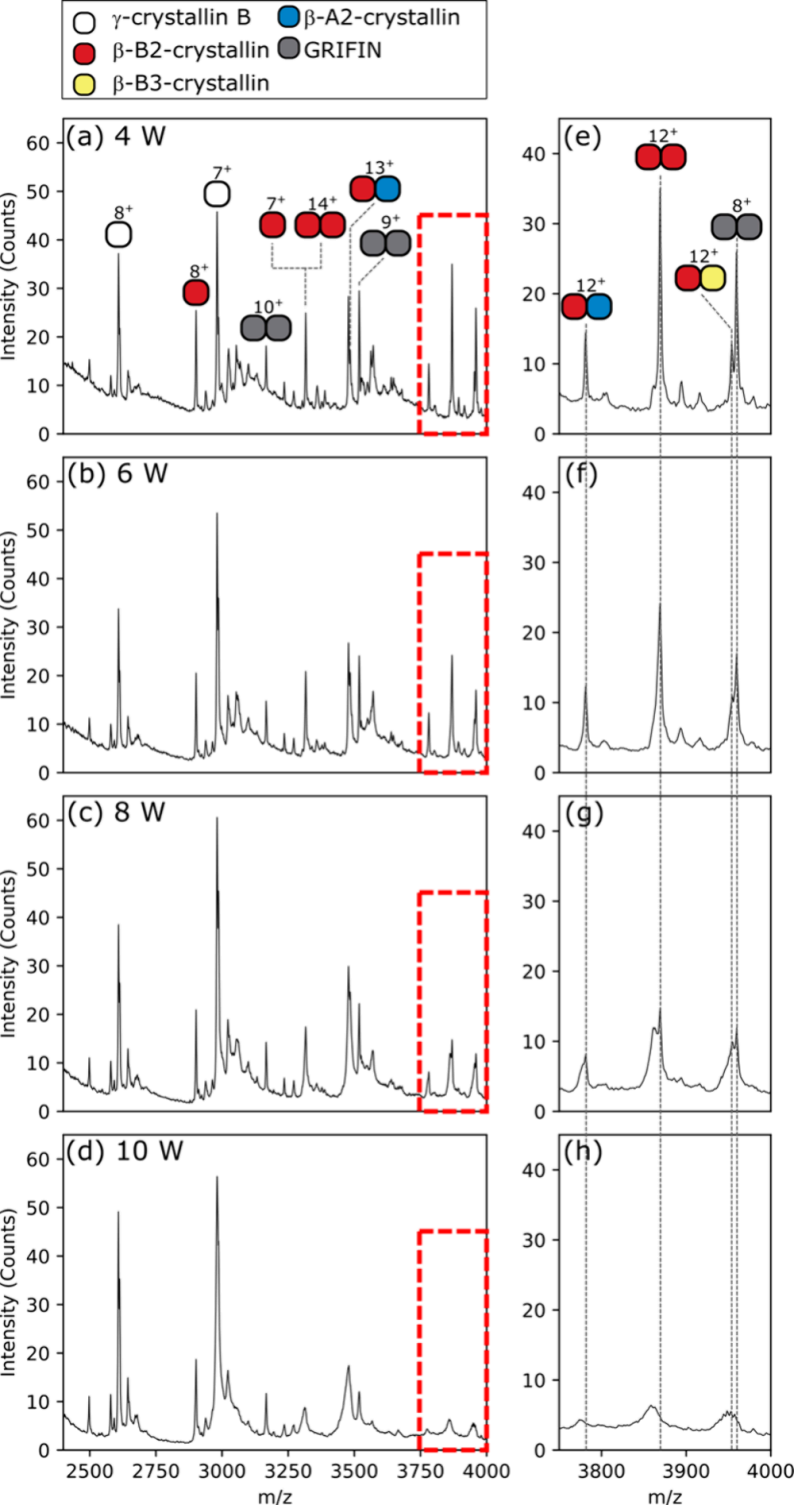
Higher laser output power is detrimental to the protein signal intensity. Panels show mass spectra for eye lens proteins at increasing laser output powers (a) 4 W, (b) 6 W, (c) 8 W, and (d) 10 W. (e–h) Cropped spectra highlight signal degradation for protein complex signals in the red dashed boxes in (a–d), respectively.

Spatially resolved nano-DESI analysis of eye lens cortex without source CID generated few protein signals, and the noise level was high (Figure 2a). With the source CID potential set to 100 V (system maximum for LTQ Velos Pro), some abundant proteins were detected, e.g., γ-crystallin S (20.86 kDa monomer, W5QH67, Figure S3 and Table S4, Supporting Information). Peak broadening due to neutral loss fragmentation with this level of source CID was not detected for these proteins, which indicates poor declustering performance with collisional activation rather than signal being absent because of protein fragmentation. Conversely, a laser output power of 6 W effectively declustered protein ions and reduced chemical noise. Intact noncovalent protein complexes of β-B2-crystallin (46.42 kDa homodimer, B2 subunit; 23.21 kDa, identification reported previously^2^), β-B2/B3-crystallin (47.43 kDa heterodimer, B3 subunit; 24.21 kDa, Figure S4 and Table S5, Supporting Information), and β-B2/A2-crystallin (45.37 kDa heterodimer, A2; 22.16 kDa, Figure S5, Supporting Information) and galectin-related interfiber protein (GRIFIN,^26^ 15.83 kDa homodimer, Figure S6, Supporting Information) were detected in multiple charge states across the *m*/*z* range. Protein ions detected with the IR-LIT system correspond to high-resolution nano-DESI MS spectra obtained using the Orbitrap Eclipse (Figure S7, Supporting Information). Laser output power exceeding 6 W resulted in noticeable broadening of the protein peak shapeowing to neutral loss fragmentation (e.g., −H_2_O). Signal intensity was simultaneously decreased (Figure 3). These effects indicate a threshold at which IR declustering is detrimental to maintaining the detected proteins and complexes intact, and instead operates in the IRMPD MS^n^ modality capable of covalent bond dissociation (Figure 3).^27^ At a 10 W laser output power, IRMPD fragmentation of crystallin dimers was evident by reduction in signal intensity and peak broadening (Figure 3d,h). Further evidence of fragmentation was detected by IRMPD MS^2^ of directly infused carbonic anhydrase. The 10+ charge state was isolated and exposed to an additional 50 ms of IR activation. Signals for *b* and *y* ions were evident at a 9 W laser output power (Figure S8, Supporting Information). For proteins and complexes analyzed from tissue here, the optimum laser output power for declustering was between 4 and 6 W with higher laser output powers leading to degradation of intact protein signals.

Native MS using nano-DESI benefits from supplemental gas-phase activation to decluster and desolvate endogenous protein ions. Nano-DESI is usually performed with flow rates in the 0.3–2 μL/min regime, far higher than the low nL/min flow rates of nanoESI commonly used for native MS.^28^ On our setup, the nano-DESI emitter has an internal diameter of approximately 75 μm, necessary to ensure aspiration of the aqueous solvent by the mass spectrometer vacuum, but this produces large initial droplets from which production of desolvated protein ions is inefficient.^29^ Even on high-performance Orbitrap and Q-TOF mass spectrometers, it has been necessary to use heated ion inlets to improve protein signal-to-noise ratio for experiments using this ion source.^1,22^ In comparison, static nanoESI for native MS is typically performed with emitters in the 1 μm internal diameter regime that enhance the production of desolvated and desalted ions through small initial droplets. The benefits of a similar IRMPD setup on an Orbitrap Eclipse mass spectrometer have already been demonstrated for declustering membrane proteins from detergent when introduced by nanoESI.^18^ The proof-of-concept nano-DESI-LIT platform indicates that the native nano-DESI performance could be improved on a range of low- and high-performance mass spectrometers by implementation of IR declustering.

## Conclusions

Native MS using nano-DESI on a linear ion trap mass spectrometer was demonstrated by integration of a 10.6 μm laser to enhance protein ion declustering. The prototype IR-LIT enabled intact mass analysis of protein complexes up to 47 kDa in molecular weight directly from brain and eye lens tissue, with protein identification confirmed by a high-resolution mass spectrometer. The proof-of-concept LIT system lacks synchronization of laser on/off, laser power, and mass spectrometer scan events, which if implemented would allow finer control of *where* ions are irradiated along the beamline and more sophisticated experiments (e.g., MS^n^). Nevertheless, the system presented here indicates that a substantial improvement can be made to protein ion declustering from the nano-DESI ion beam using IR photons. The results are relevant to the development of low- and high-performance mass spectrometers incorporating an alternative to collision-based declustering.

## Data Availability

Supplementary data supporting this research is openly available from the University of Birmingham data archive at DOI: 10.25500/edata.bham.00001201.

## Supporting Information Available

Instrument diagram, details on protein assignments, and their identification by MS/MS (PDF)
