# Supplementary material for: Infrared Photoactivation Enables nano-DESI MS of Protein Complexes in Tissue on a Linear Ion Trap Mass Spectrometer
Source: J Am Soc Mass Spectrom. 2024 Nov 28;36(1):146–52. doi: 10.1021/jasms.4c00377 (PMC11697349; doi:10.1021/jasms.4c00377)
Supplement: Supplementary file 1 — js4c00377_si_001.pdf [file js4c00377_si_001.pdf]

Supporting Information

Infrared photoactivation enables nano-DESI MS of protein complexes in tissue on a linear ion trap mass spectrometer.

Oliver J. Hale*, Todd H. Mize, Helen J. Cooper

School of Biosciences, University of Birmingham, Edgbaston, Birmingham, B15 2TT, UK.

Supporting Figures

Figure S1: IR-LIT setup diagram 3
Figure S2: Mouse brain protein ion signals: IR-LIT and orbitrap........ 4
Figure S3: nano-DESI-HCD MS2 of γ-crystallin S 6
Figure S4: nano-DESI-HCD MS2 of β-B2/B3-crystallin 8
Figure S5: nano-DESI-HCD MS2 of β-B2/A2-crystallin 9
Figure S6: nano-DESI-HCD MS2 of GRIFIN 10
Figure S7: Sheep eye lens protein ion signals: IT-LIT and orbitrap 11
Figure S8: ESI-IRMPD MS2 of carbonic anhydrase........ 12

Supporting Tables

Table S1: average intact molecular weight (MW) for non-SOD mouse brain proteins and complexes. 5
Table S2: Parameters for deconvolution with UniDec........ 5
Table S3: average intact molecular weight (MW) for metal-bound hSOD1^{G93A} complexes........ 5
Table S4: HCD sequence ions for γ-crystallin S. 7
Table S5: HCD sequence ions for β -B3-crystallin. 8

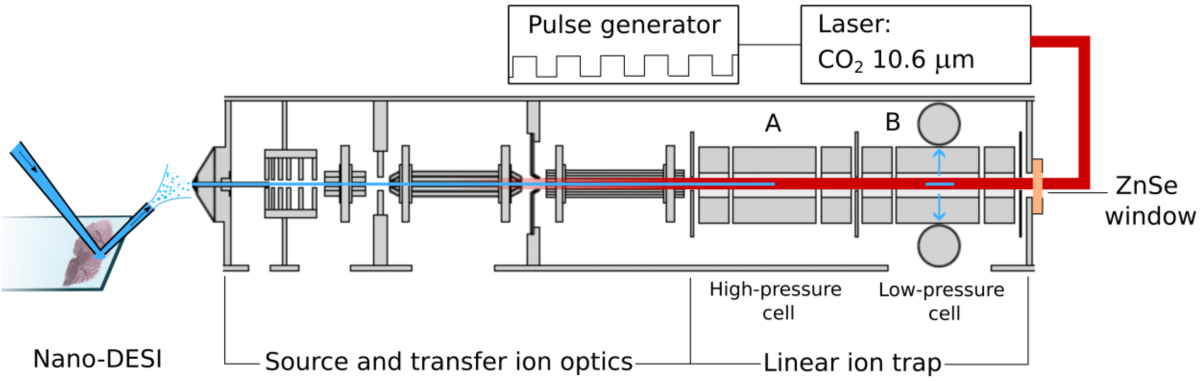

Figure S1: setup of the nano-DESI-IR-LIT. Proteins were sampled from thin tissue sections by the nano-DESI ion source and delivered as a continuous ion beam to the high-pressure cell. ***A****: Ions were accumulated for the specified injection time (IT), or until AGC target was reached. Accumulating ions were continuously irradiated by the coaxial IR laser beam. Beam power was dictated by the duty cycle of a square waveform provided by a pulse generator.* ***B****: Once IT or AGC target was reached, the ion packet was injected into the lower pressure cell for m/z analysis. Beams and components are not shown to scale.*

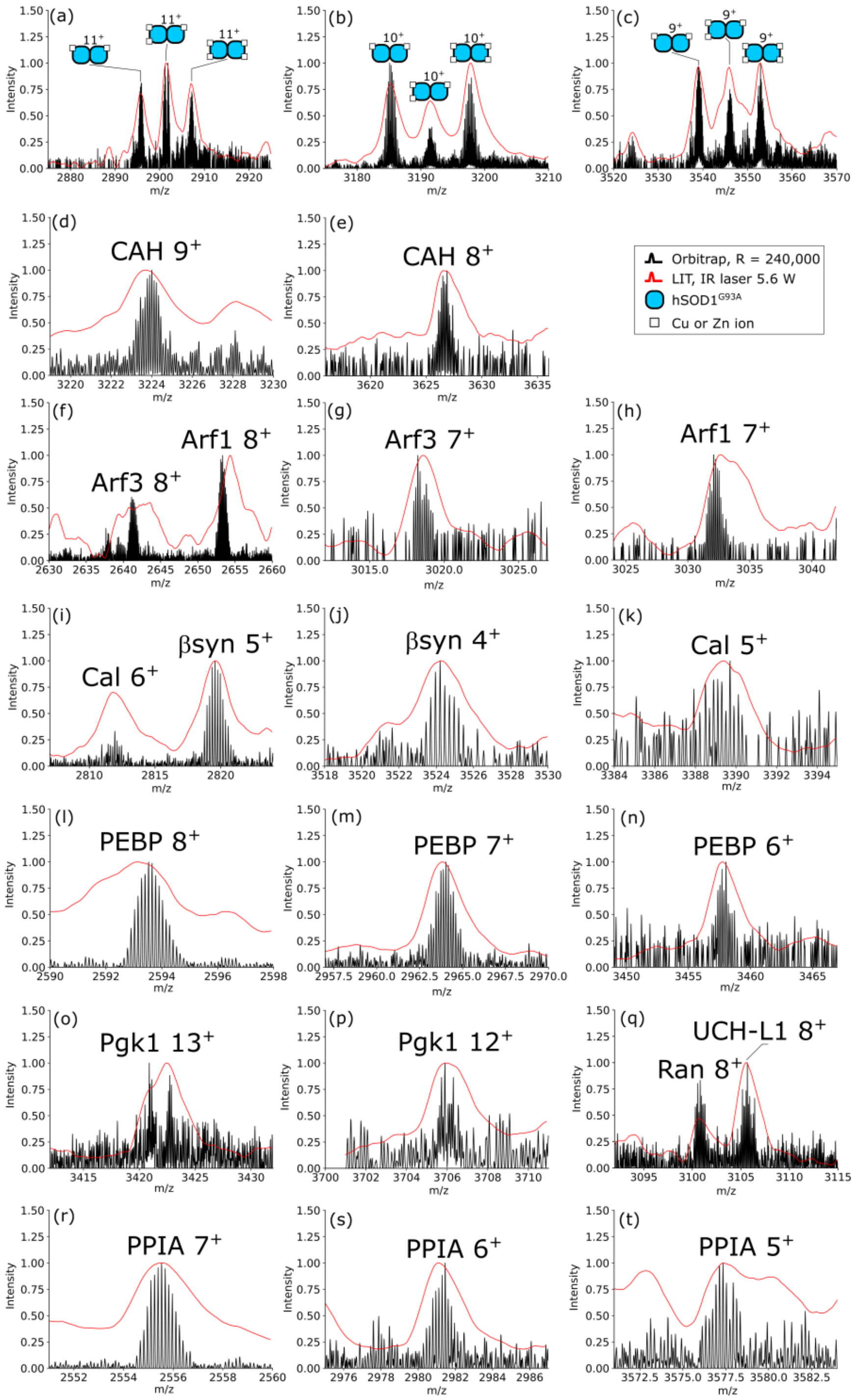

Figure S2: Comparison of peaks in nano-DESI high-resolution orbitrap MS spectra (black, R= 240,000 FWHM at m/z 200) and nano-DESI-IR-LIT MS spectra (red) obtained from the brainstem of $hSOD1^{G93A}$ transgenic mouse brain. Panels (a) – (c): The triplet of $hSOD1^{G93A}$ in metal deficient (2 and 3 metal ions) and holo (4 metal ions) in three charge states (11+, 10+ and 9+). (d) and (e): Carbonic anhydrase 2 bound to its endogenous ligand, Zn^{2+} in 9+ and 8+ charge states. (f) 8+ charge states of Arf3 and Arf 1 both bound to GDP. (g): 7+ charge state of Arf3 with GDP. (h) 7+ charge state of Arf1 with GDP. (i) 6+ and 5+ charge states of calmodulin bound to $3Ca^{2+}$ and βsyn respectively. (j) 4+ charge state of βsyn. (k) 5+ charge state of calmodulin with $3Ca^{2+}$. (l, m, n) 8+, 7+ and 6+ charge states of PEBP1. (o, p) 13+ and 12+ charge states of Pgk1. (q) 8+ charge states of Ran and UCH-L1. (r, s, t) 7+, 6+ and 5+ charge states of PPIA. Spectra are normalised. LIT spectra were baseline subtracted using mMass.[1]

Table S1: average intact molecular weight (MW) for non-SOD mouse brain proteins and complexes.

Protein	Uniprot	Ligand	PTM	LIT MW (Da)	Calculated MW (Da)
**PPIA**	P17742	N/A	-M, +N-acetyl	17882	17882
**PEBP**	P70296	N/A	-M, +N-acetyl	20740	20741
**Cal**	P0DP26	3x Ca^{2+}	-M, +N-acetyl	16868	16869
**Arf3**	P61205	GDP	-M, +N-myristoylation	21123	21124
**Arf1**	P84078	GDP	-M, +N-myristoylation	21221	21220
**Ran**	P62827	GDP, Mg^{2+}	-M, +N-acetyl	24799	24802
**UCH-L1**	Q9R0P9	N/A	N/A	24839	24838
**CAH**	P00920	Zn^{2+}	-M,+N-acetyl	29004	29008
**Pgk1**	P09411	N/A	-M, +N-acetyl	44458	44462
**βSyn**	Q91ZZ3	N/A	N/A	14096	14095

Table S2: Parameters for deconvolution with UniDec. Other parameters were left as default.

UniDec parameter	Value
**m/z range**	2600 – 3800
**Background subtraction**	Yes
**Charge range**	5 – 15
**Mass range (Da)**	20000 – 40000
**Sample Mass Every (Da)**	1
**Peak detection range (Da)**	25
**Peak detection threshold**	0.2

Table S3: average intact molecular weight (MW) for metal-bound hSOD1^{G93A} complexes.

Complex	PTM (per monomer)	Deconvoluted MW (LIT)	Deconvoluted MW (Orbitrap)	Calculated MW (Da)
**2 metal ions**	-M, +N-acetyl, disulfide	31842	31841	31841.955
**3 metal ions**	M, +N-acetyl, disulfide	31905	31905	31905.348
**4 metal ions**	M, +N-acetyl, disulfide	31967	31966	31968.741

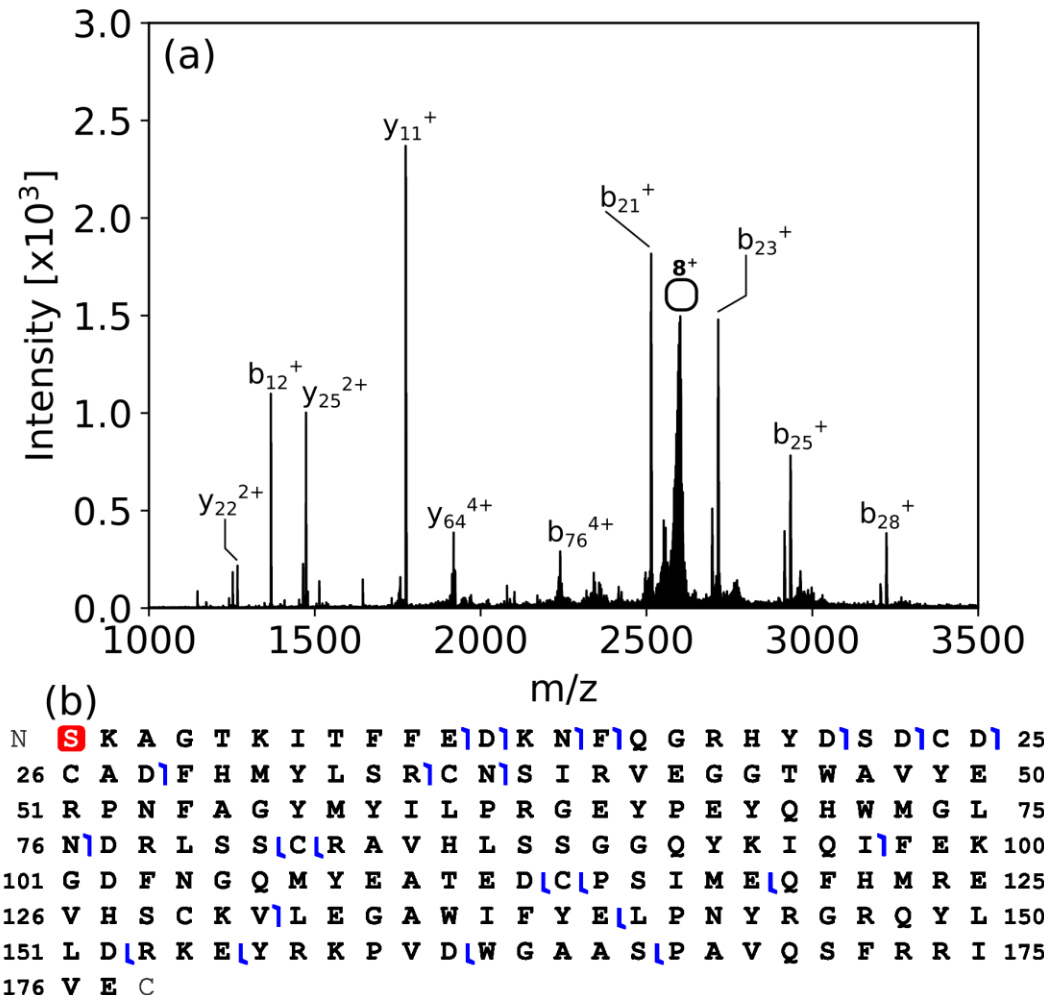

Figure S3: nano-DESI-HCD MS2 spectrum of γ-crystallin S (m/z 2608.7^{8+} ± 2.5). Example fragment ions are labelled. (b) The sequence of γ-crystallin S with fragment ions indicated.

Table S4: HCD sequence ions for γ-crystallin S.

Name	Observed Mass (Da)	Theoretical Mass (Da)	Mass Difference (ppm)
**b11**	1251.6464	1251.6499	-2.8
**b12**	1366.6652	1366.6769	-8.5
**b14**	1608.8045	1608.8147	-6.4
**b15**	1755.8994	1755.8832	9.3
**b21**	2512.1878	2512.2135	-10.2
**b23**	2714.2520	2714.2725	-7.5
**b25**	2932.2831	2932.3086	-8.7
**b28**	3221.3465	3221.3818	-11.0
**b35**	4155.7846	4155.8302	-11.0
**b37**	4372.8613	4372.8823	-4.8
**b76**	9003.9838	9004.0766	-10.3
**b97**	11303.3961	11303.2621	11.9
**b131**	15306.9036	15306.9717	-4.4
**y11**	1300.7120	1300.7251	-10.1
**y16**	1772.9177	1772.9322	-8.2
**y22**	2531.3102	2531.3397	-11.6
**y25**	2944.5656	2944.5783	-4.3
**y37**	4433.3422	4433.3621	-4.5
**y58**	7023.5435	7023.5870	-6.2
**y63**	7580.7797	7580.8389	-7.8
**y64**	7683.7869	7683.8481	-8.0
**y95**	11183.3142	11183.4947	-16.1
**y96**	11286.3656	11286.5039	-12.3

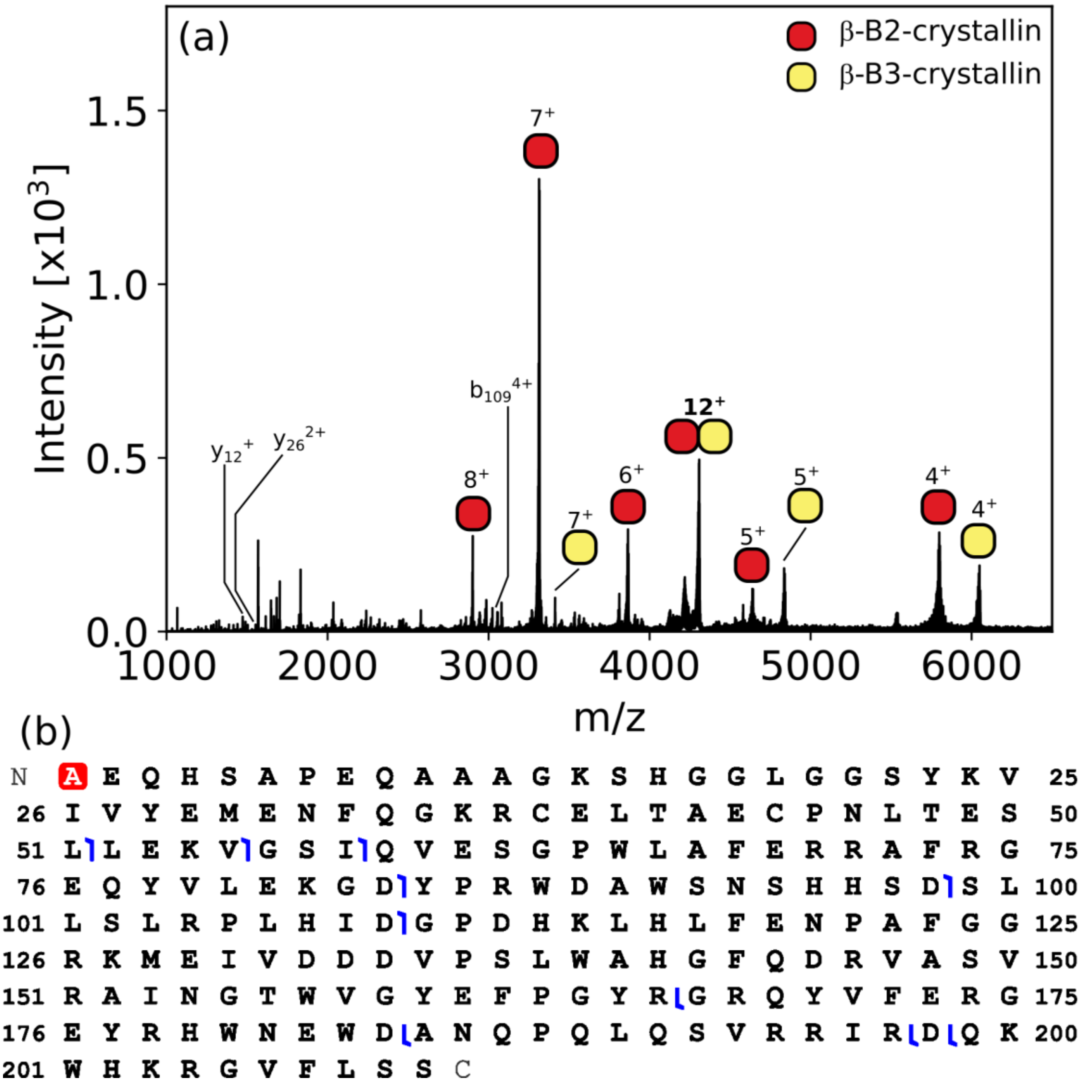

Figure S4: (a) nano-DESI-HCD MS^2 spectrum of the heterodimeric β-B2/B3-crystallin complex (m/z 4311^{11+}±2.5). (b) The sequence of b-B3-crystallin with HCD product ions indicated. Example fragments are labelled in (a).

Table S5: HCD sequence ions for β-B3-crystallin.

Ion	Observed Mass (Da)	Theoretical Mass (Da)	Error (ppm)
**b51**	5458.5524	5458.5656	-2.4
**b55**	5927.8466	5927.8556	-1.5
**b58**	6184.9719	6184.9932	-3.4
**b84**	9233.4790	9233.5137	-3.8
**b98**	10972.2060	10972.2373	-2.9
**b109**	12216.8946	12216.9614	-5.5
**y12**	1471.8053	1471.8048	0.4
**y13**	1586.8292	1586.8317	-1.6
**y26**	3133.7055	3133.7122	-2.1
**y44**	5541.7121	5541.7956	-15.1

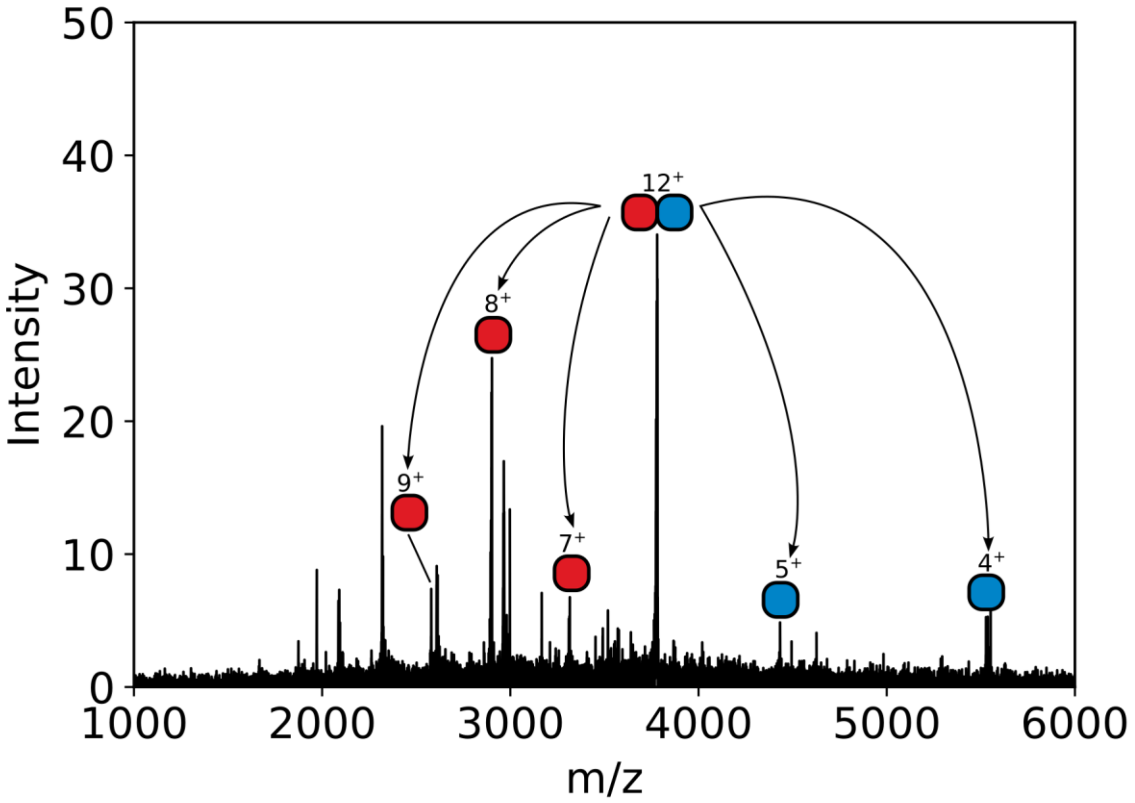

Figure S5: Nano-DESI-HCD MS² spectrum of the β-B2/A2-crystallin heterodimer. (Intact mass match only). β-crystallin A2: A0A6P7DS74

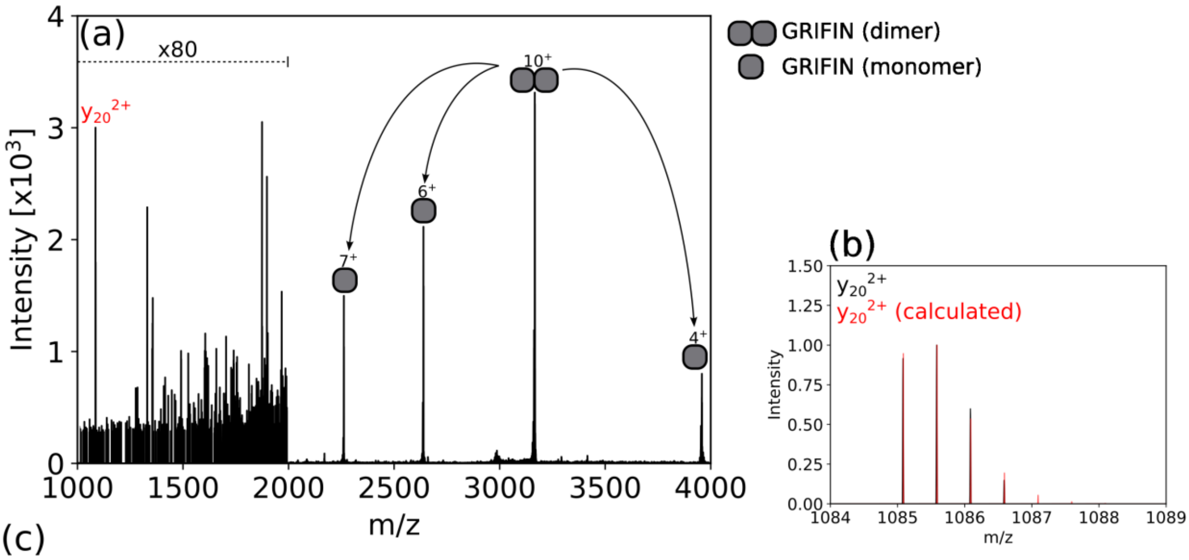

(c)

```
N   F C L Q F E A F H A G G L A P G W N L L V Q G H S   25
26  D S G E D Q F E I N F L S E T G D I V F H I K P R   50
51  F S S A T M V A N A F Q G G R W G Q E E A S S V F   75
76  P L V L G E P F E M E V S S D A E H F H V H A Q E  100
101 H K V L R F A H R H R P L A A I T R V Q V L S D H  125
126 R L A Q V E L A R K G L S W G A G G Y C
```

Figure S6: (a) nano-DESI-HCD MS2 of the abundant lens protein GRIFIN. The precursor ions dissociated to monomer signals of half the precursor mass, indicating homodimeric stoichiometry. The measured intact mass is 167 Da less than predicted by the UniProt entry (TrEMBL, October 2023) for GRIFIN (W5P4T5) potentially indicating detection of an undocumented isoform or proteoform. The fragment ions y_{20}^{+} and (b) y_{20}^{2+} were detectable and correspond to fragmentation of the aspartic acid residue most proximal to GRIFIN's C-terminus. Measurement error for y_{20}^{2+} = -5.4 ppm. b-ions were not detected, suggesting a non-canonical N-terminus. (c) The sequence for GRIFIN indicating the y_{20}^{n+} fragment.

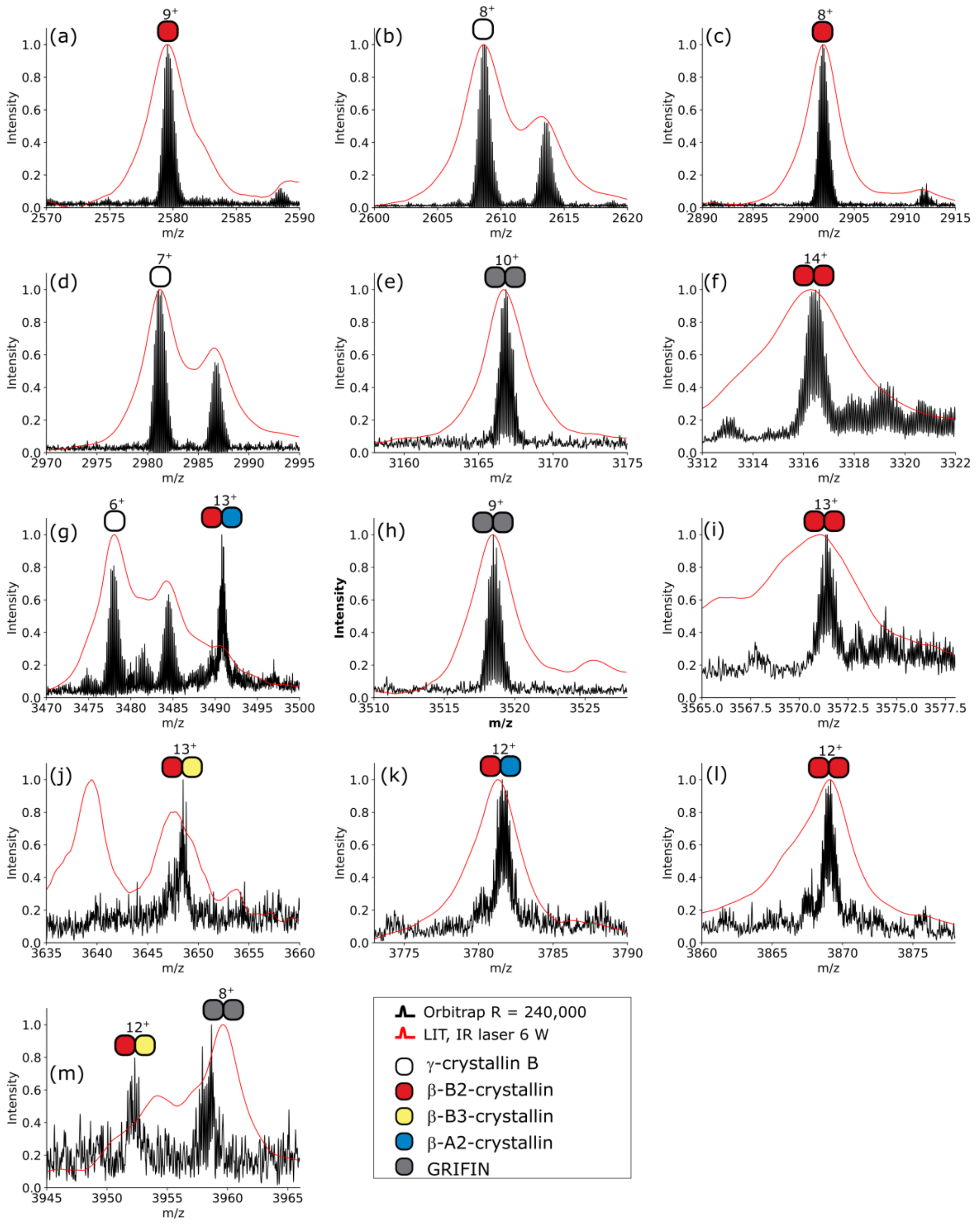

Figure S7: Comparison of peaks in nano-DESI high-resolution orbitrap MS spectra (black, R= 240,000 FWHM at m/z 200) and nano-DESI-IR-LIT MS spectra (red) obtained from the eye lens in order of ascending m/z. (a, c) monomeric β-B2-crystallin, (b, d, g) monomeric γ-crystallin, (e, h, m) homodimeric GRIFIN, (f, i, l) homodimeric β-B2-crystallin, (g, k) β-B2/β-A2-crystallin heterodimers and (j, m) β-B2/β-B3-crystallin heterodimers. Intensities are normalized. LIT spectra were baseline subtracted using mMass.[1]

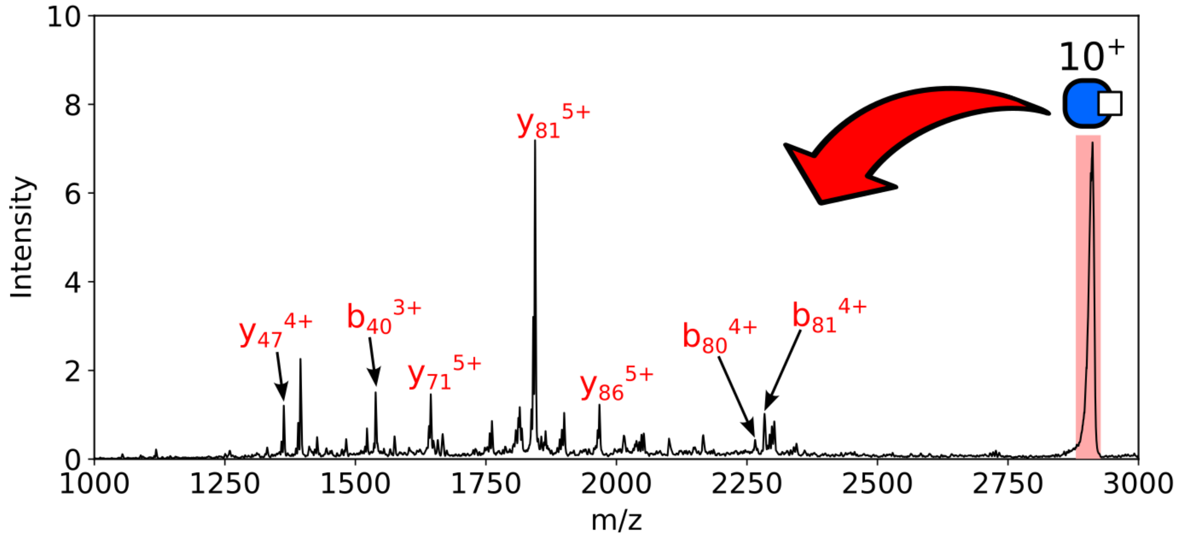

Figure S8: ESI-IRMPD MS^2 of carbonic anhydrase infused in aqueous ammonium acetate and ionised by direct infusion ESI. IR laser output power 9 W.

References

(1) Strohalm, M.; Kavan, D.; Novák, P.; Volný, M.; Havlíček, V. mMass 3: A Cross-Platform Software Environment for Precise Analysis of Mass Spectrometric Data *Anal Chem* **2010**, *82*, 4648-4651, 10.1021/ac100818g
